# Cross-organ translation of antifibrotic strategies from skin and lung fibrosis to glaucoma filtration surgery

**DOI:** 10.3389/fphar.2026.1979114

**Published:** 2026-10-26

**Authors:** Xiaolu Zhong, Guiling Zhao

**Affiliations:** 1 The First Clinical College, Guangdong Medical University, Zhanjiang, Guangdong, China; 2 Department of Ophthalmology, Affiliated Hospital of Guangdong Medical University, Zhanjiang, Guangdong, China

**Keywords:** antifibrotic drugs, cross-organ translation, drug delivery system, filtering bleb fibrosis, glaucoma filtration surgery, targeted therapy, Tenon capsule fibroblasts

## Abstract

Filtering bleb scarring after glaucoma filtration surgery (GFS) remains the leading cause of surgical failure, and current antimetabolites are limited by non-selective cytotoxicity that restricts their long-term use. Research on skin and lung fibrosis has accumulated extensive experience in antifibrotic mechanisms and therapeutic approaches. These tissues share a common transforming growth factor-beta (TGF-β)-driven program of fibroblast activation and extracellular matrix (ECM) remodeling with Tenon capsule fibroblasts, providing a theoretical basis for translating antifibrotic strategies across organs. From a cross-organ translational medicine perspective, this review systematically summarizes progress in translating antifibrotic research from skin and lung fibrosis to antiscarring therapy for GFS, focusing on four aspects: shared fibrotic mechanisms; repurposed approved drugs and emerging molecular targets; local drug delivery systems (DDS); and ophthalmology-specific translational challenges. It also highlights mechanotransduction via Yes-associated protein (YAP)/transcriptional co-activator with PDZ-binding motif (TAZ), lysyl oxidase (LOX)-mediated ECM crosslinking, and the translational potential of local sustained-release delivery in filtering blebs. Building on this foundation, we propose that multi-target combination therapy, precision local delivery, and individualized antiscarring strategies based on biomarkers such as aqueous humor TGF-β_2_ may represent important future directions for improving the long-term success rate of GFS.

## 1 Introduction

Glaucoma filtration surgery (GFS) remains the primary surgical intervention for lowering intraocular pressure when medical therapy is insufficient (Kavitha et al., 2024). Despite advances in surgical techniques and adjunctive therapies, postoperative filtering bleb fibrosis continues to be the leading cause of surgical failure, with reported 5-year failure rates of approximately 35% after primary trabeculectomy and approaching 50% in high-risk or previously failed eyes (Kavitha et al., 2024; Gedde et al., 2022; 2012). Current clinical strategies mainly rely on mitomycin C (MMC) and 5-fluorouracil (5-FU) to suppress postoperative scarring; however, both agents act via non-selective cytotoxicity, increasing the risk of complications such as thin-walled bleb, persistent hypotony, and bleb-related endophthalmitis (Yang et al., 2023; Gedde and Herndon, 2025). CAT-152, an anti-transforming growth factor-β_2_ (TGF-β_2_) monoclonal antibody, is the only molecularly targeted antifibrotic agent evaluated to date in a phase III clinical trial to prevent postoperative filtering bleb scarring. Its failure to meet the primary endpoint highlighted the complexity of fibrosis regulation and suggested that single-target strategies may be insufficient for effective fibrosis control after GF (Cat-152 0102 Trabeculectomy Study Group, 2007).

In recent years, research on hypertrophic scarring (HS) and idiopathic pulmonary fibrosis (IPF) has advanced substantially, spanning antifibrotic mechanistic insights, targeted drug development, and local delivery systems. Accumulating evidence indicates that skin, lung, and filtering bleb fibrosis share core pathomechanisms, including fibroblast activation, extracellular matrix (ECM) remodeling, mechanotransduction, and immune regulation (Sun et al., 2021; Lurje et al., 2023; Li D. J. et al., 2024). However, translating these therapeutic strategies directly to GFS remains challenging because of the unique anatomical and physiological barriers of the ocular surface (Yang and Lockwood, 2022; Ahmed et al., 2023). To date, no review has integrated shared fibrotic mechanisms, therapeutic candidates, local delivery technologies, and ophthalmology-specific translational barriers from a cross-organ perspective. To address this gap, this review summarizes recent advances in translating antifibrotic strategies from skin and lung fibrosis to antiscarring therapy after GFS across these four dimensions, and discusses future directions toward precision antifibrotic approaches.

## 2 Shared fibrotic mechanisms across skin, lung and filtering blebs

### 2.1 Conserved TGF-β/Smad signaling

TGF-β represents a central driver of fibrosis across HS, IPF, and ocular fibrotic disorders. Through Smad3-dependent signaling, TGF-β drives fibroblast-to-myofibroblast transition, induces α-smooth muscle actin (α-SMA) expression, and accelerates ECM deposition (Flanders, 2004; Shu and Lovicu, 2017). Unlike skin and lung fibrosis, where TGF-β_1_ is the predominant isoform, filtering blebs are continuously exposed to an aqueous humor environment enriched with TGF-β_2_ (Flanders, 2004; Tan et al., 2024). Studies in human conjunctival fibroblasts further demonstrated that, although both TGF-β_1_ and TGF-β_2_ regulated gene expression of major ECM proteins, only TGF-β_1_ increased three-dimensional spheroid stiffness, whereas TGF-β_2_ lacked this effect (Watanabe et al., 2023). These findings suggest that distinct TGF-β isoforms may contribute to the unique regulatory features of fibrosis in filtering blebs compared with other organs.

### 2.2 Mechanotransduction through YAP/TAZ

Both HS and IPF studies have established that matrix stiffness drives myofibroblast activation through the integrin–cytoskeleton-Yes-associated protein (YAP)/transcriptional coactivator with PDZ-binding motif (TAZ) axis, representing a key mechanotransduction mechanism that sustains fibrotic progression (Sun et al., 2021; Li D. J. et al., 2024). The filtering bleb is continuously subjected to mechanical stimulation arising from intraocular pressure fluctuations and bleb wall tension, a mechanical environment analogous to wound tension in skin and to tissue stretch in the lung, which promotes fibrosis. This mechanism has been preliminarily validated in human Tenon’s capsule fibroblasts and in animal models. SPARC, TGF-β_2_ (the dominant isoform in aqueous humor), and interstitial fluid flow have all been shown to activate YAP/TAZ signaling (Du et al., 2023; Futakuchi et al., 2018; Frömmichen et al., 2025). Furthermore, increased *in vivo* ECM stiffness promotes Tenon’s capsule fibroblast activation through integrin αvβ6 signaling (Feng et al., 2025), supporting a pivotal role for the mechanotransduction–YAP pathway in filtering bleb scarring. Beyond pathological matrix stiffening, the ocular environment is also subject to additional dynamic mechanical inputs, including ocular pulsations (Johnstone et al., 2021; Madekurozwa et al., 2021), as well as external mechanical stimuli such as postoperative ocular massage and eye rubbing (Smith et al., 2010; Turner et al., 2019; Prud’homme et al., 2023). Whether these stimuli influence bleb remodeling through YAP/TAZ remains to be established.

### 2.3 LOX-mediated ECM remodeling

Lysyl oxidase (LOX) catalyzes the covalent cross-linking of collagen and elastin, thereby driving a progressive increase in ECM stiffness (Vallet and Ricard-Blum, 2019). In skin and lung fibrosis, the sustained upregulation of LOX and lysyl oxidase-like 2 (LOXL2) is considered a critical step in the transition from reversible tissue damage to irreversible fibrotic remodeling (Poe et al., 2023). Similarly, elevated expression of LOX and LOXL2 has been detected in Tenon’s tissue and aqueous humor of patients with glaucoma, which correlates with postoperative filtering bleb outcomes (NikhalaShree et al., 2019). In addition, long-term exposure to anti-glaucoma medications may induce LOXL2 expression in Tenon’s capsule fibroblasts (Nikhalashree et al., 2022). LOX-mediated ECM crosslinking is not only a critical step in structural scar maturation but may also amplify integrin–YAP/TAZ mechanotransduction by increasing matrix stiffness, thereby forming a profibrotic positive-feedback loop. Since ECM stiffening directly impedes aqueous humor outflow, LOX inhibitors are expected to act synergistically with anti-TGF-β strategies.

### 2.4 Persistence of myofibroblasts

Persistent myofibroblast survival represents another conserved feature across these fibrotic disorders. During physiological wound repair, myofibroblasts are normally cleared via apoptosis and related mechanisms once tissue restoration and matrix remodeling are complete, thereby maintaining tissue homeostasis (Wilson et al., 2020; Merkt et al., 2021). In contrast, within HS, IPF, and Tenon’s capsule fibroblasts, sustained TGF-β stimulation triggers pro-survival pathways such as PI3K/Akt signaling, conferring resistance to apoptosis and enabling these cells to persist (Zhang et al., 2023; Cho et al., 2022; Tai et al., 2020). These persistent myofibroblasts subsequently continue to synthesize and deposit ECM, hindering the resolution of fibrotic tissue. Accordingly, targeting cell proliferation alone may be insufficient to reverse established fibrosis.

### 2.5 Immune regulation of fibrosis

Immune regulation represents another conserved component of the fibrotic network across organs. In skin and lung fibrosis, M2 macrophages secrete TGF-β and platelet-derived growth factor (PDGF), which act in concert with the Th2 cytokines IL-4 and IL-13, with the latter driving STAT6 signaling, to promote fibroblast activation and ECM deposition (Huang et al., 2023; Chao et al., 2023; Lurje et al., 2023). M2 macrophage infiltration is also evident in filtering bleb tissue, with the M2c subset recognized as a potentially predominant profibrotic subpopulation (Sacchi et al., 2025); however, direct evidence linking this specific subset to profibrotic mechanisms remains lacking.

Taken together, these findings show that skin, lung, and filtering bleb share an interconnected fibrotic network centered on TGF-β and reinforced by ECM remodeling, mechanotransduction, and immune regulation, providing a mechanistic rationale for translating antifibrotic strategies across organs (Figure 1).

**FIGURE 1:**
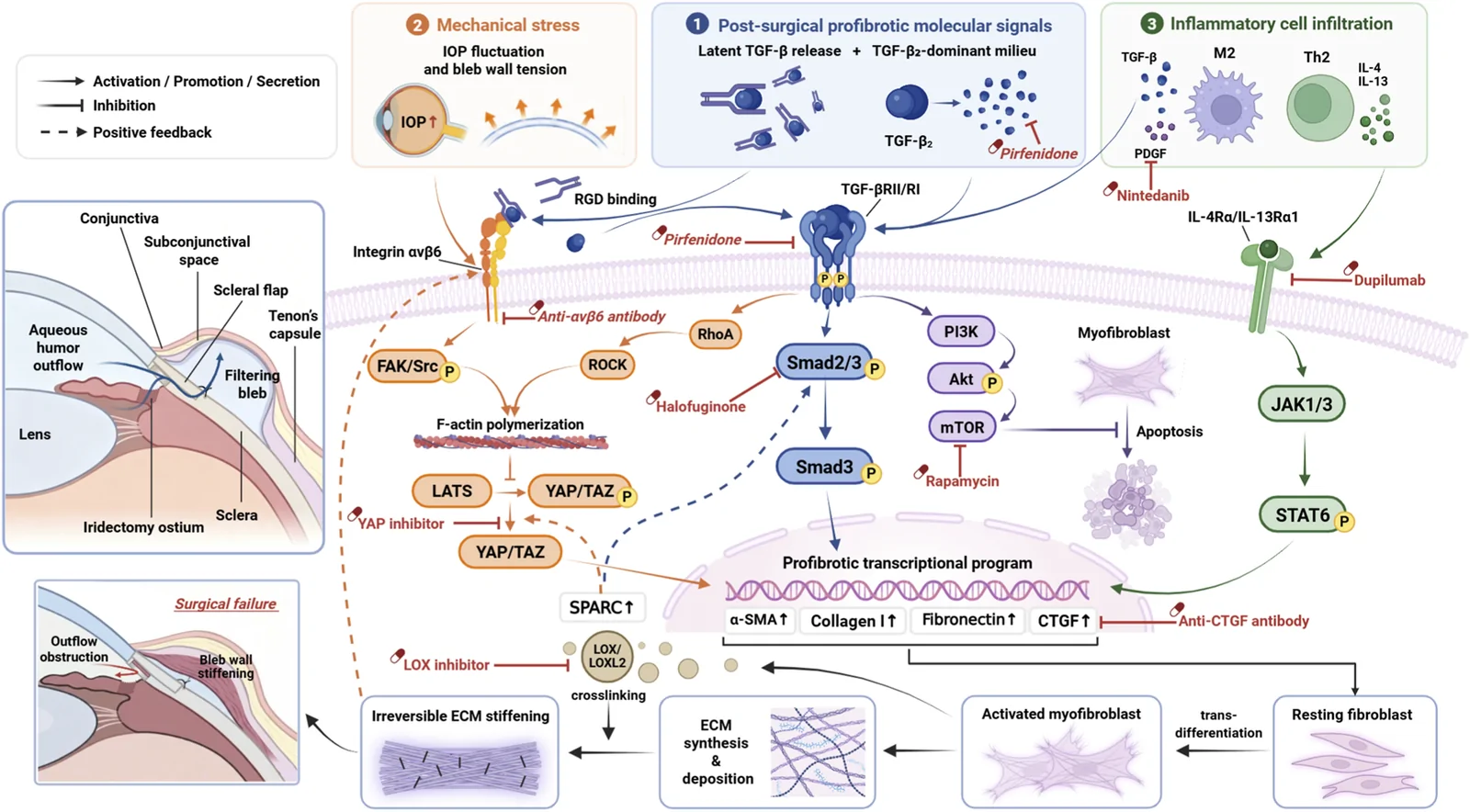
Molecular mechanisms and therapeutic targets in filtering bleb fibrosis after glaucoma filtration surgery. Surgical injury, TGF-β_2_-dominant aqueous humor, mechanical stress, and inflammatory mediators converge to drive fibroblast-to-myofibroblast transition and ECM deposition. Integrin αvβ6 links mechanical stress to latent TGF-β activation and YAP/TAZ signaling; TGF-β/Smad, YAP/TAZ, and IL-4/IL-13–JAK/STAT pathways then activate a profibrotic transcriptional program. Myofibroblast-derived SPARC further amplifies TGF-β/Smad and YAP/TAZ signaling, while LOX-mediated ECM crosslinking causes progressive stiffening and positive feedback, and PI3K/Akt/mTOR promotes myofibroblast survival by suppressing apoptosis. Red capsule symbols indicate therapeutic intervention points. Color-coded arrows denote TGF-β/Smad (blue), YAP/TAZ (orange), PI3K/Akt/mTOR (purple), and STAT6 (green) signaling. Solid arrows indicate activation, promotion, or secretion; blunt-ended lines indicate inhibition; dashed arrows indicate positive feedback. Created in https://BioRender.com.

## 3 Translational opportunities for antifibrotic therapies

Building on these shared fibrotic mechanisms, antifibrotic strategies derived from skin and lung fibrosis research are increasingly being explored for antiscarring therapy after GFS (Table 1).

**TABLE 1:** Antifibrotic strategies with translational potential for GFS.

Candidate strategy	Source field and clinical status	Target and mechanism	Ocular/GFS evidence	GFS translational stage*	Major translational barrier
Pirfenidone	Lung (IPF) (FDA-Approved)	TGF-β/Smad3, VEGF	• *In vitro* (HTFs) (Na et al., 2015) • *In vivo* (Rabbit GFS) (Kasar et al., 2021) • Safety (Canine) (Westermeyer et al., 2019)	Advanced Preclinical	Low ocular bioavailability due to corneal/scleral barriers; lack of clinical GFS trials
Nintedanib	Lung (IPF) (FDA-Approved)	RTK (VEGFR/PDGFR/FGFR)	• *In vitro* (HTFs, conjunctival endothelium) (Lin et al., 2018; Cheng et al., 2025) • *In vivo* (Rabbit GFS) (Yüksel et al., 2023)	Advanced Preclinical	Limited ocular *in vivo* data; optimal dosing and long-term intraocular safety not established
Rapamycin *(Sirolimus)*	Skin/Transplant (FDA-Approved)	mTORC1/Autophagy	• *In vitro* (hSCFs) (Wu et al., 2020) • *In vivo* (Rabbit GFS) (Tai et al., 2020; Igarashi et al., 2021)	Advanced Preclinical	Incomplete blockade of downstream 4E-BP1 axis; unvalidated in human GFS
Anti-αvβ6 mAb *(BG00011/STX-100)*	Lung (IPF) (Phase IIb Trial)	Upstream TGF-β activation	• GFS-related (HTFs) (Feng et al., 2025) • Other ocular (mouse cornea) (Blanco-Mezquita et al., 2011; Wu et al., 2019)	Early Preclinical	Failed in Phase IIb IPF trial *(acute exacerbations)*; αvβ8/TSP-1 compensation; limited GFS *in vivo* validation (rat model only)
Anti-CTGF mAb *(Pamrevlumab)*	Lung (IPF) (Phase IIb Trial)	Downstream CTGF signaling	• GFS model (Rabbit GFS) (Hassanpour et al., 2022) • Other ocular (ECM, PVR/CNV) (Wallace et al., 2013; Daftarian et al., 2019 ; 2021)	Advanced Preclinical	Failed in Phase III IPF trial *(ZEPHYRUS-1)*; single-target blockade insufficient for mature scars; poor ocular penetration
Halofuginone	Skin/Keloid (Investigational)	Midstream Smad3 phosphorylation	• GFS model (Rabbit GFS) (Kasar et al., 2021) • Other ocular (Corneal cells) (Nelson et al., 2012)	Advanced Preclinical	Narrow therapeutic window and potential toxicity; lacks human safety data; urgent need for dedicated ocular sustained-release DDS

* GFS Translational Stage was categorized as follows: Early Preclinical, initial proof-of-concept phase validated primarily through *in vitro* cell models or non-GFS, ocular tissue models; Advanced Preclinical, achieved robust *in vivo* disease-model validation in GFS (e.g., rabbit trabeculectomy models) with established efficacy metrics, awaiting formal ocular clinical trial initiation.

Abbreviations: CNV, choroidal neovascularization; CTGF, connective tissue growth factor; DDS, drug delivery system; ECM, extracellular matrix; FGFR, fibroblast growth factor receptor; GFS, glaucoma filtration surgery; hSCFs, human subconjunctival fibroblasts; HTFs, human Tenon’s capsule fibroblasts; IPF, idiopathic pulmonary fibrosis; mAb, monoclonal antibody; mTORC1, mechanistic target of rapamycin complex 1; PDGFR, platelet-derived growth factor receptor; PVR, proliferative vitreoretinopathy; RTK, receptor tyrosine kinase; TGF-β, transforming growth factor-beta; TSP-1, thrombospondin-1; VEGF, vascular endothelial growth factor; VEGFR, vascular endothelial growth factor receptor.

### 3.1 Repurposing approved drugs for antifibrotic therapy

Compared with developing entirely new ophthalmic agents, drug repurposing offers shorter development timelines and well-established safety profiles, making it a practical pathway for cross-organ translation. The most extensively investigated candidates to date include pirfenidone, nintedanib, and rapamycin, each of which has undergone translational validation from skin or lung fibrosis to Tenon’s capsule fibroblasts and animal models, although they differ in mechanism of action, therapeutic advantages, and translational maturity.

#### 3.1.1 Pirfenidone

Pirfenidone is a U.S. Food and Drug Administration (FDA)–approved antifibrotic agent for IPF. By inhibiting TGF-β/Smad signaling, it attenuates ECM deposition and has demonstrated clear efficacy in both HS and IPF (Knighton et al., 2025; Coentro et al., 2021). In human Tenon’s capsule fibroblasts, pirfenidone suppresses cell migration and α-SMA expression, with lower cytotoxicity than MMC (Na et al., 2015). In rabbit models of trabeculectomy and glaucoma drainage device implantation, topical pirfenidone effectively inhibits filtering bleb fibrosis, with efficacy largely comparable to MMC (Kasar et al., 2021; Jung and Park, 2016). Furthermore, long-term topical administration in a canine model demonstrated no apparent corneal toxicity (Westermeyer et al., 2019). Pirfenidone also suppresses vascular endothelial growth factor (VEGF)-mediated neovascularization (Liu et al., 2017). Clinically, anti-VEGF monoclonal antibodies (e.g., bevacizumab) have been investigated as adjuncts in glaucoma filtration surgery, often in combination with MMC, as well as in drainage devices and as an adjunctive therapy for neovascular glaucoma (Zhou et al., 2023; He et al., 2024; Ramji et al., 2025). These agents have also been shown to reduce the requirement for postoperative needling (Ramji et al., 2025). Given this clinical context, the anti-VEGF property of pirfenidone suggests its potential utility in neovascular glaucoma. Nonetheless, existing studies have primarily employed eye drop administration, with short ocular surface residence time and limited local drug exposure remaining major challenges.

#### 3.1.2 Nintedanib

Nintedanib is a multi-target tyrosine kinase inhibitor (TKI) that simultaneously blocks vascular endothelial growth factor receptor (VEGFR), platelet-derived growth factor receptor (PDGFR), and fibroblast growth factor receptor (FGFR) signaling, selectively induces apoptosis of senescent fibroblasts, and inhibits fibroblast activation (Cutolo et al., 2021; Cho et al., 2022). In human Tenon’s capsule fibroblasts, nintedanib blocks TGF-β_1_-induced fibrotic responses by inhibiting Smad2/3, p38 MAPK, and ERK1/2 signaling (Lin et al., 2018), while suppressing VEGF-induced proliferation and tube formation in conjunctival vascular endothelial cells (Cheng et al., 2025). In a rabbit trabeculectomy model, nintedanib prolonged filtering bleb survival and reduced tissue fibrosis scores, outperforming 0.2% MMC without apparent toxicity (Yüksel et al., 2023). Currently, however, available ocular evidence remains largely confined to *in vitro* studies and a single animal study.

#### 3.1.3 Rapamycin

Rapamycin, an mTOR pathway inhibitor, exerts antifibrotic effects partly through autophagy activation and has been shown to attenuate skin fibrosis in mouse models of systemic sclerosis (Wu et al., 2020; Sekiguchi et al., 2025). In human conjunctival fibroblasts, rapamycin inhibits TGF-β_1_-induced α-SMA and collagen expression (Wu et al., 2020). In rabbit trabeculectomy models, it has also been shown to reduce early postoperative collagen deposition and scar formation (Igarashi et al., 2021), while promoting fibroblast apoptosis and prolonging filtering bleb survival (Tai et al., 2020). Furthermore, an injectable rapamycin-loaded hydrogel achieved sustained drug release in the subconjunctival space and effectively suppressed fibrosis in a rat model, presenting a promising translational strategy for local ocular delivery (Sun et al., 2024). However, rapamycin exhibits only limited inhibition of TGF-β_1_-induced mTORC1/4E-BP1 signaling, which may be attributable to its allosteric inhibitory mechanism that does not fully block downstream 4E-BP1 signaling (Jiayu et al., 2024). Hence, its antifibrotic efficacy as monotherapy remains constrained, leaving clear room for further optimization.

### 3.2 Emerging targeted antifibrotic strategies

Although these agents have validated the feasibility of cross-organ antifibrotic therapy, their broad-spectrum mechanisms of action may limit therapeutic precision. Consequently, research focus has increasingly shifted toward precise interventions at key molecular nodes within fibrotic signaling networks. Anti-αvβ6 integrin antibodies, anti-connective tissue growth factor (CTGF) monoclonal antibodies, and halofuginone exemplify current strategies targeting distinct nodes of the fibrotic cascade—upstream latent TGF-β activation, downstream effector signaling, and the intermediate Smad3 pathway, respectively. All three have been mechanistically validated in skin and lung fibrosis and show preliminary translational potential in ocular anti-scarring applications.

#### 3.2.1 Anti-αvβ6 integrin antibody

Integrin αvβ6 is a transmembrane receptor predominantly expressed on epithelial cells that triggers the fibrotic cascade in skin and lung fibrosis by activating latent TGF-β (L-TGF-β) (Notni, 2022; Bellani et al., 2024). In tendinopathy models, this receptor was further shown to activate TGF-β in response to mechanical loading, with αvβ6 knockout significantly attenuating fibrosis (Wang X. et al., 2022), positioning it as a critical node bridging mechanotransduction and fibrogenesis.

Within the filtering bleb, intraocular pressure fluctuations and bleb wall tension similarly generate persistent mechanical stress, which may drive local TGF-β activation through a shared mechanism. Corneal wound healing studies have established that αvβ6 is required for basement membrane regeneration and contributes to the rapid initiation of fibrosis (Blanco-Mezquita et al., 2011; Wu et al., 2019). More importantly, Feng et al. recently demonstrated for the first time that younger patients exhibit greater matrix stiffness and more severe fibrosis, using human Tenon’s capsule fibroblasts and a rat model of GFS. Specifically, increased matrix stiffness upregulates αvβ6 expression and promotes fibroblast activation, whereas αvβ6 knockdown significantly attenuates filtering bleb scarring (Feng et al., 2025). Thus, anti-αvβ6 integrin antibodies hold promise for precise intervention upstream in the fibrotic cascade, avoiding the immune-related adverse effects of direct TGF-β neutralization while offering greater target specificity than broad-spectrum antifibrotics.

Despite its strong mechanistic rationale, the clinical translation of anti-αvβ6 strategies still warrants caution. Indeed, the anti-αvβ6 monoclonal antibody BG00011 was prematurely terminated in a phase IIb clinical trial for IPF due to adverse events (Raghu et al., 2022), raising concerns that systemic αvβ6 blockade may excessively suppress the physiological functions of TGF-β. In GFS, however, subconjunctival or intracameral administration could enable localized delivery directly to the Tenon’s capsule while minimizing systemic exposure, potentially providing a more feasible translational path for ophthalmic applications.

#### 3.2.2 Anti-CTGF antibody

As a key downstream effector in the TGF-β signaling pathway, connective tissue growth factor (CTGF) plays a central mediating role in fibroblast activation, ECM synthesis, and fibrotic progression (Yanagihara et al., 2022). In muscle fibrosis models, CTGF blockade has been shown to reduce fibrosis and improve tissue function (Lambi et al., 2023; Connolly et al., 2023); similar antifibrotic efficacy has been observed in pulmonary fibrosis models (Yanagihara et al., 2022). Compared with direct TGF-β antagonism, targeting CTGF is expected to preserve the anti-inflammatory and immunomodulatory functions of TGF-β, theoretically offering a broader therapeutic window.

This strategy is supported by ocular studies. Specifically, aqueous humor CTGF levels are significantly higher in patients with pseudoexfoliation syndrome and glaucoma than in controls, and the degree of elevation correlates with disease severity (Ho et al., 2005). In human trabecular meshwork and lamina cribrosa cells, the anti-CTGF antibody FG-3019 has been shown to significantly block the upregulation of α-SMA, collagen IA1, and fibronectin induced by aqueous humor from glaucoma patients (Wallace et al., 2013). Rabbit trabeculectomy models have shown that subconjunctival injection of an anti-CTGF antibody achieved antifibrotic efficacy comparable to that of MMC, without the corneal toxicity commonly associated with MMC (Hassanpour et al., 2022). In addition, anti-CTGF antibodies have demonstrated antifibrotic effects in models of proliferative vitreoretinopathy and choroidal neovascularization (Daftarian et al., 2019; 2021), suggesting that CTGF may represent a common therapeutic target across multiple ocular fibrotic disorders.

However, the anti-CTGF antibody failed to meet its primary endpoint in the phase III ZEPHYRUS-1 trial for IPF (Raghu et al., 2024), suggesting that single-target blockade of CTGF may be limited by compensatory mechanisms within the fibrotic network. Compared with systemic administration, local delivery during GFS may achieve higher drug exposure at the target site, whereas combination strategies such as co-targeting VEGF may further enhance antifibrotic efficacy.

#### 3.2.3 Halofuginone

In the TGF-β/Smad signaling pathway, Smad3 predominantly mediates the transcription of profibrotic genes, whereas Smad2 is more involved in maintaining tissue homeostasis (Flanders, 2004). Halofuginone is a selective Smad3 inhibitor that suppresses TGF-β_1_-induced expression of α-SMA and type I procollagen while reducing migration and matrix contraction in keloid fibroblasts (Marty et al., 2021). Compared with broad-spectrum TGF-β inhibition, selective blockade of Smad3 is expected to suppress fibrosis while preserving certain physiological TGF-β functions.

In ocular studies, halofuginone has similarly demonstrated antifibrotic effects. In human corneal fibroblasts, it dose-dependently downregulates Smad3 expression, thereby suppressing TGF-β-induced α-SMA and fibronectin expression, as well as type I collagen deposition (Nelson et al., 2012). A rabbit trabeculectomy model further confirmed that intraoperative local application of halofuginone reduced TGF-β and bFGF expression and suppressed fibroblast proliferation to an extent comparable to MMC, without apparent corneal toxicity (Kasar et al., 2021). Moreover, halofuginone inhibited bFGF-induced corneal neovascularization in a mouse corneal micropocket model (Elkin et al., 2000), indicating antifibrotic and antiangiogenic potential. However, current evidence remains limited to *in vitro* experiments and a single animal model, and its long-term ocular surface safety as well as the impact of sustained Smad3 inhibition on normal corneal repair remain to be further validated. Its physicochemical properties may also complicate direct ocular administration, potentially necessitating encapsulation strategies.

Overall, emerging molecular targets promise to enhance the precision of antifibrotic therapy, but single-target strategies remain vulnerable to compensatory and redundant mechanisms within the complex fibrotic network. Furthermore, most evidence remains at the cellular or preclinical stage, with key barriers to clinical translation including local delivery efficiency, long-term safety, and tissue specificity.

### 3.3 Modulating the immune microenvironment

Beyond direct targeting of the TGF-β pathway, immune cells and the inflammatory microenvironment also contribute to fibrotic progression. Therefore, modulating the postoperative immune microenvironment may be an important complementary strategy to optimize anti-scarring efficacy further.

IL-4 and IL-13 are key cytokines that drive both Th2 immune and M2 macrophage polarization, with IL-13 recognized as a common mediator of fibrosis across organs (Lurje et al., 2023). In keloids and systemic sclerosis, IL-4/IL-13 signaling directly promotes fibroblast activation, collagen synthesis, and ECM deposition through STAT6 phosphorylation (Chao et al., 2023; Huang et al., 2023), suggesting this pathway is one of the core drivers of cutaneous fibrosis. Clinically, dupilumab, an IL-4Rα antagonist, has been shown to downregulate Th2-related and fibrosis-related pathways in prurigo nodularis, further supporting the potential of targeting the IL-4/IL-13/STAT6 axis to reduce systemic fibrotic burden (Bao et al., 2024). However, its efficacy in keloids exhibits marked heterogeneity (Bitterman et al., 2024), indicating that the antifibrotic effects of IL-4/IL-13 pathway inhibition may depend on the baseline state of the local immune microenvironment.

Notably, in the treatment of atopic dermatitis, a subset of patients develops dupilumab-associated ocular surface disease (DAOSD), which has been linked to a shift in ocular surface immune balance toward Th1/Th17 responses following IL-4/IL-13 blockade (Thormann et al., 2024; Cassagne et al., 2025). A similar immune shift is also observed in systemic Th17-associated diseases (Bridgewood et al., 2022). Moreover, the risk of ocular surface disease with selective IL-13 blockade does not differ significantly from that observed with dupilumab, suggesting that IL-13 may play a predominant role in maintaining ocular surface homeostasis (Fan et al., 2026).

Collectively, these findings suggest that local modulation of the IL-4/IL-13 axis may help reshape the postoperative immune microenvironment and mitigate fibrosis following filtration surgery, although potential compensatory immune shifts, particularly Th17 activation, warrant careful consideration. Thus, beyond the TGF-β/Smad pathway, achieving an appropriate balance between antifibrotic efficacy and ocular surface immune homeostasis may represent an important direction for the future optimization of anti-scarring strategies in filtration surgery.

Anti-scarring therapy for filtering blebs is gradually shifting from broad-spectrum suppression toward mechanism-based precision intervention, driven by approved drug repurposing and precise targeting of key molecular pathways and the immune microenvironment. However, the clinical benefit of these strategies ultimately depends on achieving sustained, stable, and safe drug exposure within the filtering bleb. Therefore, developing local drug delivery systems adapted to the ocular microenvironment represents a critical step toward further clinical translation.

## 4 Translating local drug delivery strategies to filtering blebs

Drug efficacy in preventing postoperative scarring depends not only on the intrinsic pharmacological properties of the agent but also on its local exposure concentration and retention time within the target tissue. The physicochemical properties of certain agents, such as halofuginone, may complicate ocular formulation and require encapsulation strategies. Additionally, following GFS, rapid tear turnover, blinking, and the ocular surface barrier restrict the precorneal residence time of conventional eye drops to less than 1 min (Yang and Lockwood, 2022). Local drug delivery systems (DDS) thus serve as a critical bridge toward the clinical translation of antifibrotic strategies. Meanwhile, advances in topical sustained-release technologies for skin diseases have established a design paradigm centered on local enrichment, sustained release, and targeted delivery, which may provide valuable insights for optimizing filtering bleb anti-scarring therapies (Figure 2).

**FIGURE 2:**
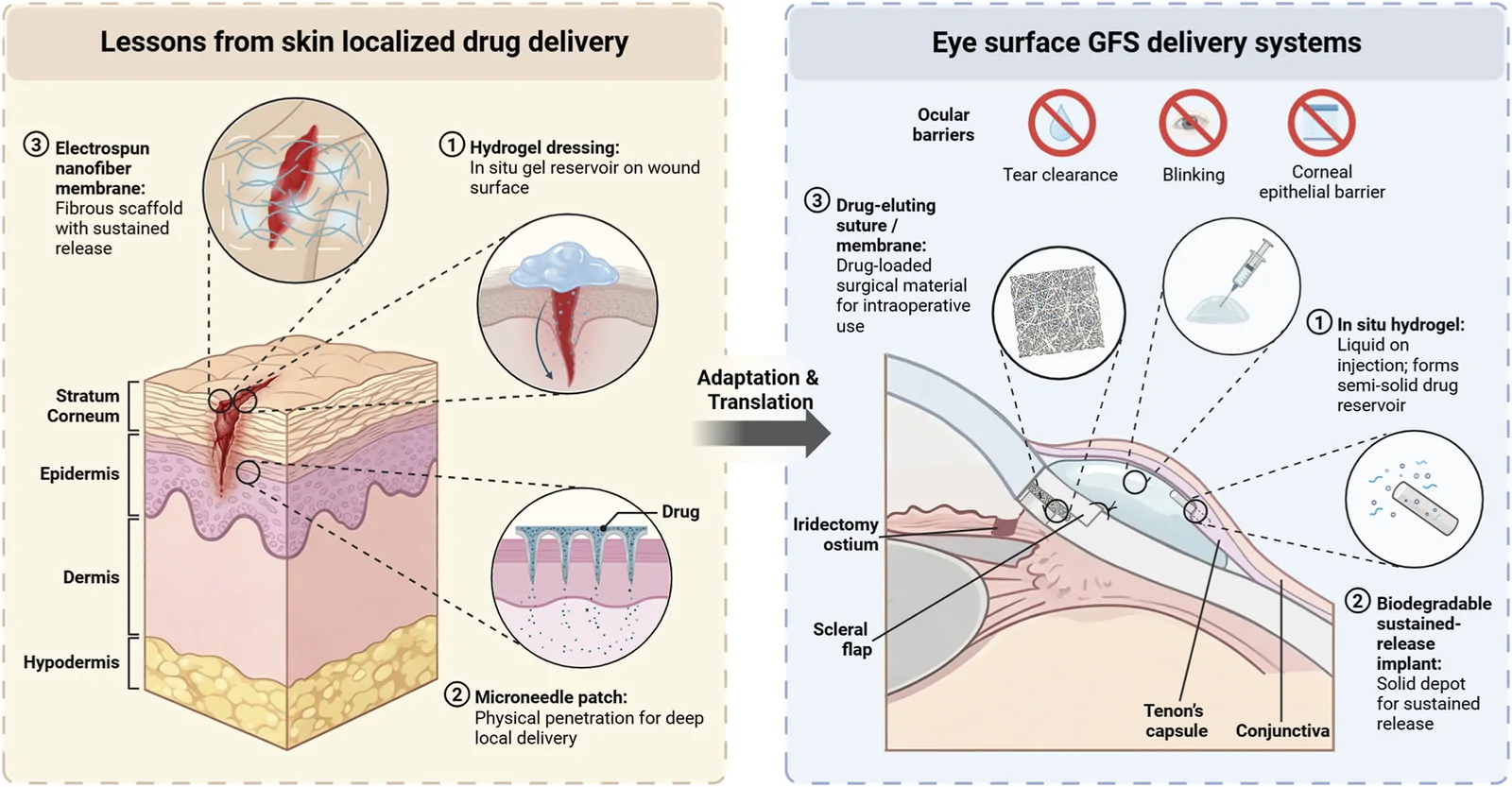
Adaptation of localized drug delivery strategies from skin to glaucoma filtration surgery. Localized drug delivery systems developed for skin provide a conceptual basis for designing antifibrotic delivery strategies for glaucoma filtration surgery. In skin, hydrogel dressings act as *in situ* drug reservoirs at the wound surface, microneedle patches enable physical penetration for deep local delivery, and electrospun nanofiber membranes provide sustained drug release. Following adaptation to the ocular environment, these strategies must account for ocular barriers and clearance mechanisms, including tear clearance, blinking, and the corneal epithelial barrier. Accordingly, hydrogel-based systems are adapted as injectable *in situ* hydrogels that form local drug reservoirs; the physical penetration principle of microneedles is translated into subconjunctival injection and biodegradable sustained-release implants; and electrospun nanofibers are adapted as drug-eluting sutures or membranes. Created in https://BioRender.com.

### 4.1 Lessons from skin drug delivery

Dermal DDS are engineered to bypass the stratum corneum barrier, facilitating high local drug accumulation and sustained release at the site of fibrosis (Kar et al., 2022). Representative strategies include microneedles, which generate microchannels to deliver drugs directly into the skin, enabling minimally invasive, controlled delivery and superior local bioavailability (Lyu et al., 2023; Mbituyimana et al., 2023); hydrogels, serving as both moist wound dressings and drug reservoirs with stimuli-responsive release capabilities (Farokhi et al., 2022; Gade et al., 2024); electrospun nanofibers, whose microstructures mimic the ECM to modulate release kinetics and have been loaded with pirfenidone for hypertrophic scar treatment (Jiang et al., 2023; Tottoli et al., 2023); and bioadhesive patches, designed for extended drug delivery via prolonged tissue adhesion (Chung et al., 2023). Despite variations in their underlying mechanisms, these approaches share a common goal: maintaining high, prolonged drug exposure at the site of fibrosis—a principle that is highly pertinent to antifibrotic therapy for filtering blebs.

### 4.2 Barriers unique to ocular delivery

Compared with the skin, ocular surface drug delivery is constrained by distinct physiological barriers, including continuous tear turnover, nasolacrimal drainage, and corneal epithelial tight junctions. Most eye drops are rapidly cleared by the tears within 15–30 s, resulting in an ocular bioavailability typically below 5% (Yang and Lockwood, 2022; Ahmed et al., 2023). Besides, the cornea’s tri-layered ‘lipid–aqueous–mucin’ sandwich structure further restricts transcorneal penetration (Wang and Pang, 2023).

Wang et al. proposed that although the ocular surface barrier restricts drug bioavailability, it also provides a physiological interface that can be exploited to design strategies for enhancing local drug retention (Wang and Zhang, 2023). To overcome these barriers, ocular surface delivery systems have evolved strategies that use bioadhesive formulations and *in situ* gels to prolong ocular surface retention against tear washout (Billowria et al., 2023; Ahmed et al., 2024), alongside penetration enhancers and nanocarriers to facilitate drug transport across corneal and conjunctival epithelial barriers (Wang and Pang, 2023; Santos et al., 2025). In essence, the design paradigm has shifted from “breaching the stratum corneum” in skin DDS to “resisting washout—prolonging retention—facilitating penetration” on the ocular surface, offering vital guidance for the development of local sustained-release systems for filtering blebs.

### 4.3 Drug delivery strategies for filtering blebs

The unique anatomical features and clearance dynamics of the ocular surface necessitate an adaptive translation of skin DDS technologies. Specifically, the physical penetration mechanism of microneedles has evolved into subconjunctival injections and sustained-release implants, while the sustained-release concept of hydrogels has developed into thermosensitive or ion-responsive *in situ* gels. Similarly, the localized delivery strategy of electrospun nanofibers is now embodied in drug-eluting sutures and drug-loaded fiber membranes. Guided by this translational framework, three ocular drug delivery routes evaluated in GFS are summarized below.

#### 4.3.1 *In situ* gels

The key advantage of *in situ* gels is that they are liquid at injection and, after subconjunctival administration, respond to physiological temperature or tear ionic stimuli to form a semisolid drug depot, enabling sustained drug release. Tu et al. demonstrated that an MMC-loaded PTMC–F127–PTMC thermosensitive hydrogel maintained functional filtering blebs more effectively than intraoperative MMC-soaked sponge application in a hypertensive monkey model, representing the only reported validation of a sustained-release DDS for GFS in a primate model to date (Tu et al., 2023). Sun et al. developed an injectable hydrogel that promoted autophagy via the PI3K/AKT/mTOR pathway and continuously released rapamycin to attenuate subconjunctival fibrosis in rats (Sun et al., 2024). RGD-containing peptides had previously been investigated as antiscarring agents in GFS, with Avila et al. reporting improved bleb formation and survival following repeated postoperative subconjunctival injections in a rabbit model (Avila et al., 1998). More recently, Lv et al. engineered a self-assembled RGD peptide hydrogel that serves dual functions as a mechanical support and drug carrier, successfully suppressing Tenon’s fibroblast activation by downregulating YAP (Lv et al., 2025). Furthermore, Wang et al. developed an oxidized hyaluronic acid–decorin hydrogel that exerts anti-scarring effects by sequestering TGF-β1, introducing an active sequestration strategy distinct from conventional sustained-release approaches (Wang R. et al., 2022). Extending this concept to dynamic control, Shi et al. reported a light-triggered adhesive hydrogel offering the potential for noninvasive, postoperative control of drug-release kinetics (Shi et al., 2026).

#### 4.3.2 Sustained-release implants

Biodegradable sustained-release microparticles and implants placed beneath the scleral flap or into the sub-Tenon’s space form localized drug depots for prolonged, site-specific delivery. Van Mechelen et al. compared MMC-loaded GFS implants made of polycaprolactone (PCL) and poly(lactic-co-glycolic acid) (PLGA) and found that PLGA degraded more completely and elicited a milder foreign-body response (Van Mechelen et al., 2023). Lee et al. developed an MMC sustained-release formulation that achieved 30 weeks of intraocular pressure (IOP) control in a rabbit model, whereas the MMC solution group maintained control for only 6.5 weeks (Lee et al., 2024). Zhang et al. incorporated 5-FU into mesoporous poly(lactic acid) (PLA) to prepare a sustained-release tablet, providing continuous drug release following a single subconjunctival implantation and effectively inhibiting TGF-β and CTGF expression (Zhang et al., 2025). Qin et al. leveraged machine learning to optimize an antibody-eluting implant, pointing to a promising direction for future DDS design (Qin et al., 2026).

#### 4.3.3 Drug-eluting surgical materials

Suturing the scleral flap is an essential step in trabeculectomy. Loading antifibrotic agents directly onto sutures or ocular surface films allows for site-specific drug release while avoiding tear washout and aqueous humor dilution. Wu et al. developed a PLLA/RGD/MMC electrospun nanofibrous membrane that achieved sustained MMC release for over 25 days in a rabbit GFS model, yielding postoperative bleb morphology and function superior to those obtained with conventional MMC sponge application (Wu et al., 2025). Ma et al. designed a thermoresponsive PLA fibrous membrane for glaucoma drainage devices (GDD), wherein localized heating triggers fiber shape recovery and drug release, enabling on-demand dynamic modulation of drug delivery (Ma et al., 2025).

These three strategies are not mutually exclusive; combining them may yield synergistic drug delivery across spatial and kinetic dimensions. Incorporating sustained-release microparticles into *in situ* gels could achieve dual-release kinetics via gel filling plus long-term microparticle release, whereas combining drug-coated sutures with *in situ* gels could extend anti-scarring coverage across multiple anatomical layers.

### 4.4 Clinical proof of concept

Local sustained-release strategies have established a preliminary foundation for clinical translation. The Ologen collagen matrix exerts its anti-scarring effect by maintaining the filtration channel and guiding orderly fibroblast growth (De Oliveira and Ferreira, 2020). In a randomized controlled trial, Ologen demonstrated a 12-month surgical success rate comparable to MMC (92.6% vs. 92.3%) and a similar safety profile (Wlaź et al., 2019). Of note, some glaucoma specialists currently use Ologen in clinical practice (Romera Romero et al., 2022). Beyond single-carrier systems, drug-loaded amniotic membranes incorporating 5-FU–PLGA nanoparticles or lyophilized fibrin scaffolds have both been shown to prolong filtering bleb survival (Li et al., 2012; Hu et al., 2015), underscoring the strong translational potential of combining bioactive scaffolds with sustained-release carriers.

In sum, the technical feasibility of local DDS for anti-scarring therapy in GFS has been preliminarily validated. Achieving synergistic optimization between precision targets and precision delivery systems may represent an important future research direction.

## 5 Discussion

### 5.1 Barriers to clinical translation

Although local sustained-release delivery prolongs drug exposure within the filtering bleb, the bleb presents a unique microenvironment and functional requirements distinct from those of the skin and lungs. Thus, these antifibrotic strategies require dedicated ophthalmic consideration of the aqueous humor microenvironment, optical safety, and preclinical models and evaluation systems before translation into clinical practice.

#### 5.1.1 The aqueous humor microenvironment

Aqueous humor undergoes continuous turnover at approximately 2–3 μL/min (Sunderland and Sapra, 2023), resulting in sustained dilution and clearance of drugs administered subconjunctivally or into Tenon’s capsule—a stark contrast to the relatively static interstitial fluid environment of the skin and lung. Given the approximately 20- to 50-fold higher ascorbic acid levels in aqueous humor than in plasma (Garg and Rewri, 2023), the local drug microenvironment may be uniquely modulated. Crucially, while skin and lung fibrosis are primarily driven by TGF-β_1_, the aqueous humor is dominated by TGF-β_2_; this isoform disparity could account for the variable efficacy of TGF-β-targeted interventions across tissues. Moreover, the eye’s immune-privileged status further distinguishes the ocular microenvironment from that of the skin and lung.

#### 5.1.2 Optical and safety requirements

Unlike dermal and pulmonary systems, ocular DDS must reconcile optical transparency with tissue safety. This mandates strictly submicron particle sizes to minimize light scattering (Jeong et al., 2025; Lee et al., 2026), a carrier refractive index that approximates that of aqueous humor (∼1.336) to preserve visual quality, and degradation products that exert no corneal endothelial or retinal toxicity. Previous studies demonstrate that silica and titanium dioxide nanoparticles can induce retinal cell damage (Park et al., 2021), whereas mesoporous silica materials containing tetrasulfide bonds may cause vitreous opacification and corneal injury (Li J. et al., 2024).

#### 5.1.3 Limitations of preclinical models

The ultimate goal of anti-scarring therapy for filtering blebs is to preserve aqueous humor outflow. Consequently, unlike cutaneous applications that rely on morphological assessments such as the Vancouver Scar Scale (VSS) (Choo et al., 2021), ophthalmology employs a functional evaluation system centered on aqueous humor outflow preservation and intraocular pressure (IOP) control (Ahmadzadeh et al., 2026). Anterior-segment OCT parameters, including bleb height, wall thickness, and internal reflectivity, have emerged as robust objective correlates of bleb function and significant predictors of surgical success (Tan et al., 2023; 2025). Critically, most preclinical evaluations still rely on rabbit trabeculectomy models. Given the higher aqueous humor turnover in rabbit eyes (≈3.5 vs. 2.75 μL/min in humans) (Goh et al., 1989; Brubaker, 1991) and a more pronounced fibrotic response, these models may limit the predictive value of candidate antifibrotic strategies in humans. In addition, there is currently no universally standardized set of outcome measures for GFS, with substantial heterogeneity in the criteria used to define surgical failure across studies (Rabiolo et al., 2024; 2025; Servillo et al., 2026).

### 5.2 Future perspectives

In summary, while research on skin and lung fibrosis has provided a substantial translational foundation for antifibrotic therapy in GFS, most evidence remains preclinical (*in vitro* and animal), leaving a substantial gap before cross-organ strategies reach clinical application. Bridging this gap will require not only further efficacy and safety evaluations, but also proactive strategies to address formulation reproducibility, manufacturing quality control, regulatory requirements, and treatment costs, as well as standardized GFS outcome measures and a clear pathway from advanced preclinical models to clinical trials.

Future research should advance along three principal avenues. First, further elucidation of filtering-bleb–specific fibrotic mechanisms is needed, particularly key networks such as YAP/TAZ-mediated mechanotransduction, LOX-driven ECM remodeling, and immune microenvironment–fibroblast interactions, to clarify tissue-specific regulatory pathways that distinguish bleb fibrosis from skin and lung fibrosis. Second, given the redundancy of fibrotic signaling networks, multi-target combination therapies may suppress pathways more effectively, while integrating localized sustained-release delivery systems could optimize drug exposure and minimize systemic adverse effects. However, although combination strategies have been explored in GFS (Andrés-Guerrero et al., 2021; Swogger et al., 2021; Ramji et al., 2025), no standardized protocols or clinical trials have yet been established for precision multi-target antifibrotic therapies targeting distinct fibrotic pathway nodes, and their synergistic mechanisms and optimal dosing regimens await rigorous validation. Third, refining efficacy evaluation by incorporating biomarkers such as aqueous humor TGF-β2 levels and quantitative bleb imaging features, and validating candidate strategies through standardized animal models and prospective clinical studies, would be a critical step toward translating cross-organ antifibrotic strategies into clinical practice. Finally, cross-organ translation may be bidirectional. Strategies applied in ophthalmology, such as RGD peptides used in GFS, may also inform the development of antifibrotic strategies for skin and lung fibrosis, suggesting a potential reverse translational pathway.
